# Supplementary material for: Dynamic transition of current-driven single-skyrmion motion in a room-temperature chiral-lattice magnet
Source: Nat Commun. 2021 Nov 24;12:6797. doi: 10.1038/s41467-021-27073-2 (PMC8613223; doi:10.1038/s41467-021-27073-2)
Supplement: Supplementary file 1 — Supplementary Information [file 41467_2021_27073_MOESM1_ESM.pdf]

Supplementary Information

Dynamic transition of current-driven single-skyrmion motion in a room-temperature chiral-lattice magnet

Licong Peng[1]*, Kosuke Karube[1], Yasujiro Taguchi[1], Naoto Nagaosa[1,2], Yoshinori Tokura[1,2,3], and Xiuzhen Yu[1]*

[1]RIKEN Center for Emergent Matter Science (CEMS), Wako, Japan.

[2]Department of Applied Physics, University of Tokyo, Bunkyo-ku, Japan.

[3]Tokyo College, University of Tokyo, Bunkyo-ku, Japan.

*Corresponding authors, e-mail: licong.peng@riken.jp; yu_x@riken.jp.

The PDF includes:

Supplementary Notes 1-4

Supplementary Figures 1-4

Supplementary Note 1: $Co_9Zn_9Mn_2$-based micro-device

The micro-device consists of a (001) $Co_9Zn_9Mn_2$ thin rectangular plate that was thinned from a bulk crystal with a focused ion beam (FIB) system. The left and right sides of the plate were connected to two Pt leads via using tungsten (W) deposition. The top and bottom edges were additionally padded by amorphous carbon layers, as shown in the Supplementary Figs. 1**a**-**c**. Supplementary Figs. 1**d**-**e** outline *x*-*z* and *y*-*z* cross-sections taken along the dashed lines in Supplementary Fig. 1**b**. The resistivity of $Co_9Zn_9Mn_2$ (~0.2 mΩ·cm)[1] is several orders of magnitude lower than that of amorphous carbon. Hence we assume that virtually all the electric current flows through $Co_9Zn_9Mn_2$.

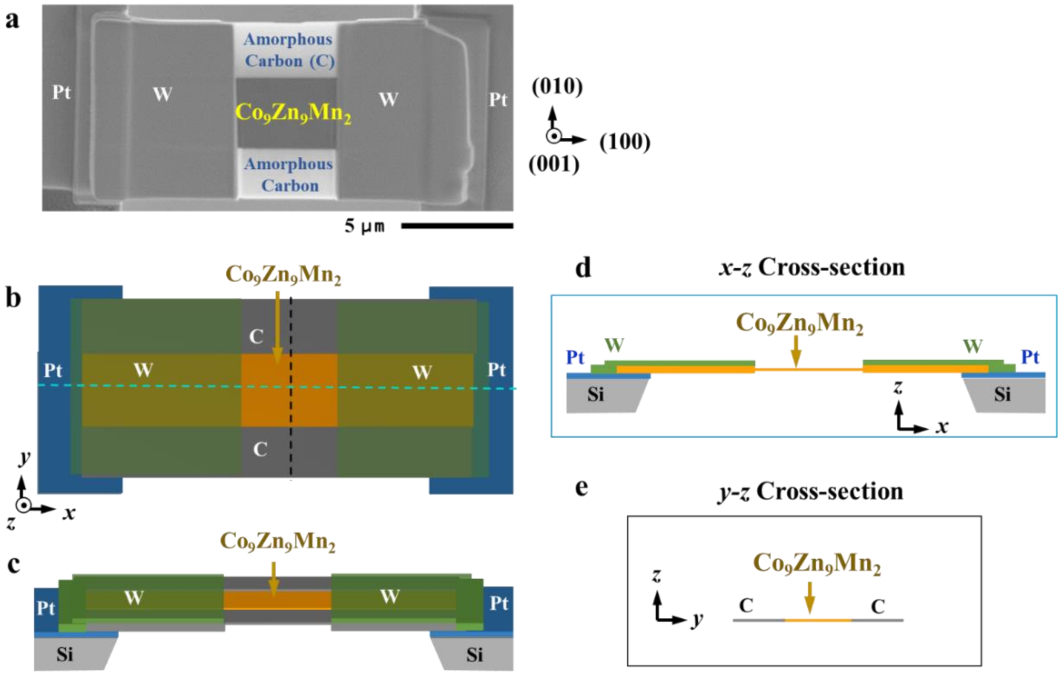

Supplementary Figure 1 | $Co_9Zn_9Mn_2$-based micro-device: (**a**) Scanning electron microscopy image. (**b**-**c**) Schematics of top and side views. (**d**-**e**) Schematics of *x*-*z* and *y*-*z* cross-sections taken along the dashed lines in (**b**).

Supplementary Note 2: Single skyrmion creation

Supplementary Fig. 2 presents the creation of a single skyrmion by the pulsed current and magnetic field. First, a conical state is formed at a magnetic field of −40 mT at room temperature (Supplementary Fig. 2**a**). A metastable skyrmion lattice is generated by a pulsed current with a density of -1.5×10^{10} A m^{-2} and a relatively large pulse duration of 300 μs while keeping the −40 mT magnetic field (Supplementary Fig. 2**b**). We then increase the field value to −450 mT to convert the skyrmion lattice into isolated skyrmions (Supplementary Fig. 2**c**). Finally, we decrease the field to −80 mT and slightly wobble its direction to ensure that only one skyrmion remains as circled in Supplementary Fig. 2**d**.

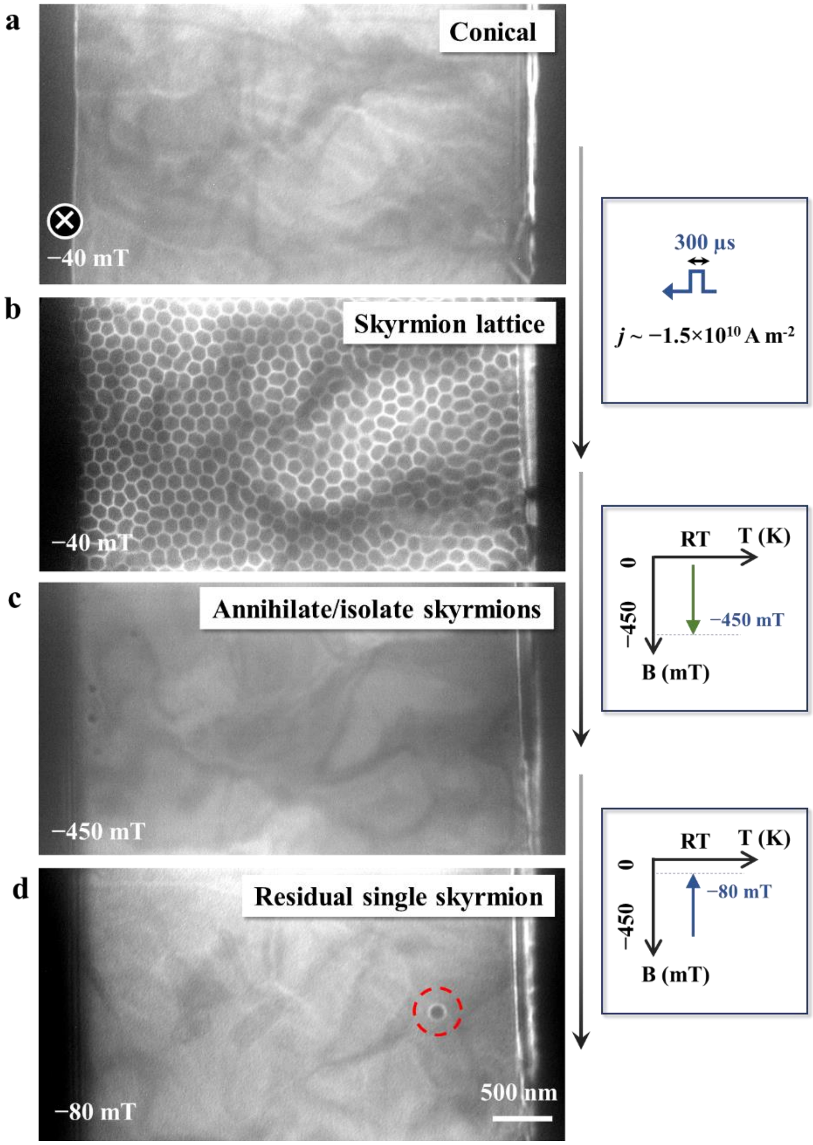

Supplementary Figure 2 | Creation of a single skyrmion at room temperature. (**a-d**) L-TEM images with the experimental protocol at the right side: (**a**) A conical state obtained at −40 mT. (**b**) A metastable skyrmion lattice generated by a 300 μs current pulse with a density of $j = -1.5\times10^{10}$ A m^{-2} while keeping the −40 mT field. (**c**) Annihilation of most skyrmions by raising the field to −450 mT. (**d**) A single skyrmion is left after decreasing the field to −80 mT and slightly wobbling its direction.

Supplementary Note 3: Magnetic induction field of $N_{sk} = -1$ skyrmion and the current-driven motion

Supplementary Figure 3**a** shows the magnetic induction field map of the $N_{sk} = -1$ skyrmion at +50 mT, extracted from under- and over-focus L-TEM images using the transport of intensity equation (TIE)[2]. The core of this skyrmion points downwards, opposite to the magnetic field, whereas the magnetization at the periphery points upwards, along the field direction (see Supplementary Fig. 3**b**). In Supplementary Figs. 3**c**-**g**, we track the Hall motion when the pulsed current flows from left to right with a density of 5.26×10^{10} A m^{-2}. The skyrmion trajectory, from the bottom-right side to the upper-left side, is reversed compared with that under a pulsed current flowing from right to left in Figs. 3**f**-**j** in the main text.

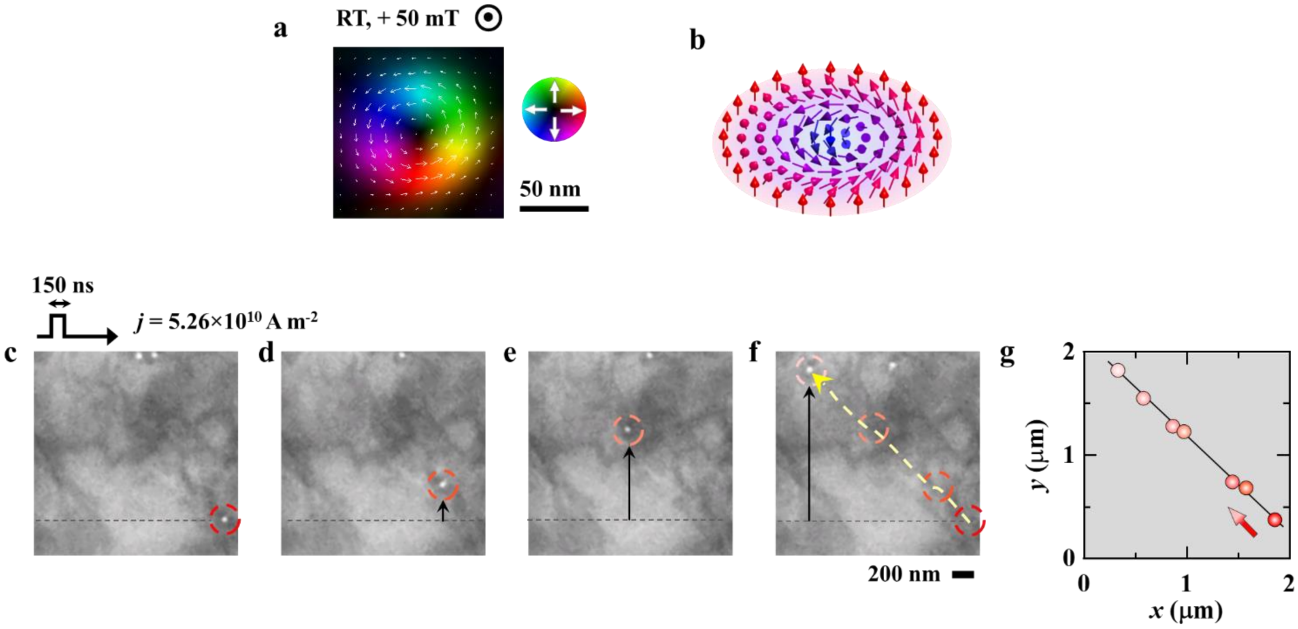

Supplementary Figure 3 | Motion tracking of the single skyrmion with $N_{sk} = -1$. (**a**) Magnetic induction field map of a metastable single skyrmion obtained at +50 mT. (**b**) Schematic of skyrmion with $N_{sk} = -1$. (**c**-**f**) Over-focus L-TEM images showing the single-skyrmion Hall motion stimulated by 150-ns current pulses with a density of $j = 5.26\times10^{10}$ A m^{-2} at +50 mT and room temperature. (**g**) Summary of

the skyrmion locations from (**c-f**) showing the reversed trajectory compared with that in Figure 3 in the main text.

Supplementary Note 4: L-TEM observation of N_{sk} = +1 skyrmion Hall motion at several current densities

In Supplementary Fig. 4, we show the L-TEM images of single-skyrmion motion at current densities j of -5.3×10^{10} A m^{-2} (Supplementary Figs. 4**a-e**), -6.82×10^{10} A m^{-2} (Supplementary Figs. 4**f-j**), and -7.57×10^{10} A m^{-2} (Supplementary Figs. 4**k-o**), respectively. Each skyrmion displacement in Supplementary Figs. 4**e**, 4**j**, and 4**o** is stimulated by a single 150-ns pulse. With increasing the current density, the skyrmion displacement increases, indicating an increase in the skyrmion velocity. Meanwhile, the skyrmion displacement is relatively uniform and linear, as exemplified by Supplementary Fig. 4**o** obtained at a higher current density of $j = -7.57\times10^{10}$ A m^{-2}. The skyrmion Hall angle, estimated by the slope of the trajectory ($\frac{\Delta y}{\Delta x}$), is ~36° at $j = -5.3\times10^{10}$ A m^{-2}, ~28° at $j = -6.82\times10^{10}$ A m^{-2}, and ~29° at $j = -7.57\times10^{10}$ A m^{-2}.

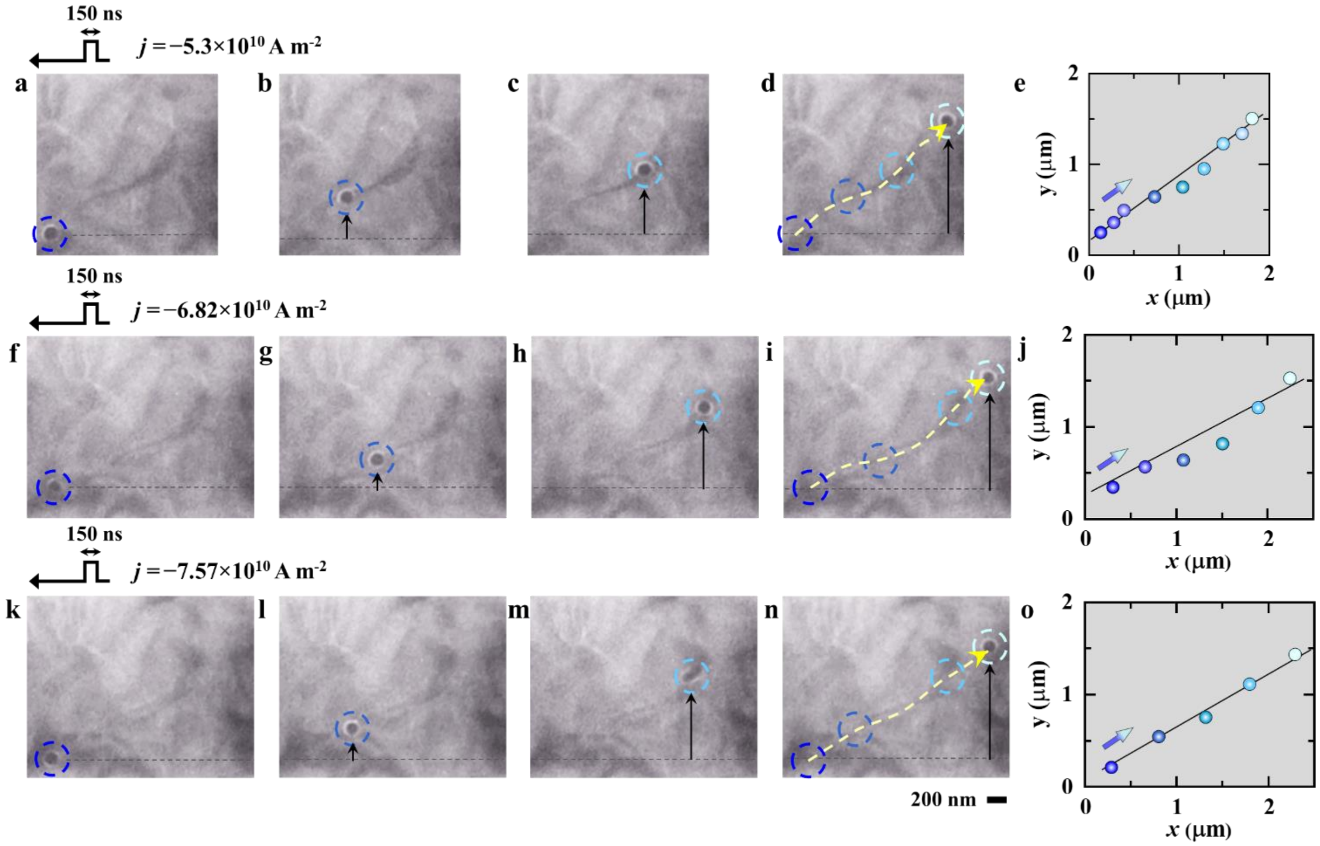

Supplementary Figure 4 | Single-skyrmion Hall motion at different current densities. (**a-d**, **f-i**, **k-n**) L-TEM images showing the single-skyrmion Hall motion and (**e**, **j**, **o**) summary of the skyrmion locations at current densities of (**a-d**) $j = -5.3\times10^{10}$ A m^{-2}, (**f-i**) $j = -6.82\times10^{10}$ A m^{-2}, and (**k-n**) $j = -7.57\times10^{10}$ A m^{-2}, respectively.

References

1. Tokunaga, Y. *et al.* A new class of chiral materials hosting magnetic skyrmions beyond room temperature. *Nat. Commun.* **6**, 7638 (2015).

2. Ishizuka, K. & Allman, B. Phase measurement of atomic resolution image using transport of intensity equation. *J. Electron Microsc.* **54**, 191–197 (2005).
