# Supplementary material for: Dynamic transition of current-driven single-skyrmion motion in a room-temperature chiral-lattice magnet
Source: Nat Commun. 2021 Nov 24;12:6797. doi: 10.1038/s41467-021-27073-2 (PMC8613223; doi:10.1038/s41467-021-27073-2)
Supplement: Supplementary file 6 — Description of Additional Supplementary Files [file 41467_2021_27073_MOESM6_ESM.pdf]

Title: Supplementary Movie 1:
Description: Single-skyrmion motion (N_{sk} = +1) induced by 150-ns electric current pulses with a density of j = −6.06 ×10^{10} A m^{-2}.

Title: Supplementary Movie 2:
Description: Single-skyrmion motion (N_{sk} = +1) induced by 150-ns electric current pulses with a density of j = 6.32 ×10^{10} A m^{-2}.

Title: Supplementary Movie 3:
Description: Single-skyrmion motion (N_{sk} = +1) induced by 150-ns electric current pulses with a density of j = −5.05 ×10^{10} A m^{-2}.

Title: Supplementary Movie 4:
Description: Single-skyrmion motion (N_{sk} = −1) induced by 150-ns electric current pulses with a density of j = −4.82 ×10^{10} A m^{-2}.
